# Sense of presence and anxiety during virtual social interactions between a human and virtual humans

**DOI:** 10.7717/peerj.337

**Published:** 2014-04-03

**Authors:** Nexhmedin Morina, Willem-Paul Brinkman, Dwi Hartanto, Paul M.G. Emmelkamp

**Affiliations:** 1 Department of Clinical Psychology, University of Amsterdam, Amsterdam, The Netherlands; 2 Interactive Intelligence Group, Delft University of Technology, Delft, The Netherlands; 3 King Abdulaziz University, Jeddah, Saudi Arabia

**Keywords:** Virtual reality therapy, Presence, Anxiety, Social phobia

## Abstract

Virtual reality exposure therapy (VRET) has been shown to be effective in treatment of anxiety disorders. Yet, there is lack of research on the extent to which interaction between the individual and virtual humans can be successfully implanted to increase levels of anxiety for therapeutic purposes. This proof-of-concept pilot study aimed at examining levels of the sense of presence and anxiety during exposure to virtual environments involving social interaction with virtual humans and using different virtual reality displays. A non-clinical sample of 38 participants was randomly assigned to either a head-mounted display (HMD) with motion tracker and sterescopic view condition or a one-screen projection-based virtual reality display condition. Participants in both conditions engaged in free speech dialogues with virtual humans controlled by research assistants. It was hypothesized that exposure to virtual social interactions will elicit moderate levels of sense of presence and anxiety in both groups. Further it was expected that participants in the HMD condition will report higher scores of sense of presence and anxiety than participants in the one-screen projection-based display condition. Results revealed that in both conditions virtual social interactions were associated with moderate levels of sense of presence and anxiety. Additionally, participants in the HMD condition reported significantly higher levels of presence than those in the one-screen projection-based display condition (*p* = .001). However, contrary to the expectations neither the average level of anxiety nor the highest level of anxiety during exposure to social virtual environments differed between the groups (*p* = .97 and *p* = .75, respectively). The findings suggest that virtual social interactions can be successfully applied in VRET to enhance sense of presence and anxiety. Furthermore, our results indicate that one-screen projection-based displays can successfully activate levels of anxiety in social virtual environments. The outcome can prove helpful in using low-cost projection-based virtual reality environments for treating individuals with social phobia.

## Introduction

Virtual reality exposure therapy (VRET) integrates real-time computer graphics, body tracking devices, visual displays and other sensory inputs to immerse individuals in computer-generated virtual environments. Thereby, it constructs the perception of an interactive, three-dimensional world. The therapeutic goals in VRET are based on treatment strategies used in behaviour therapy. Patients with anxiety disorders are treated in virtual worlds that resemble feared real life situations. Accordingly, the used virtual worlds must elicit anxiety in order to enable systematic exposure to feared stimuli within a contextually relevant situation.

VRET can make the control of exposure elements more manageable than exposure *in vivo* since the stimuli evoking anxiety can be easier changed and manipulated by the therapist. VRET most often uses a head-mounted display (HMD) or an advanced computer automatic virtual environment (CAVE) in order to increase the sense of presence in VRET (i.e., the extent to which virtual reality worlds feel realistic to participants). It has been argued that the higher the sense of presence in VRET, the better the activation of the anxiety. Yet, efforts to increase the sense of presence have not resulted in better treatment outcomes (Powers & Emmelkamp, 2008). A recent meta-analysis revealed that the association between sense of presence and perceived anxiety within VRET depends on the disorder. Whereas large correlations were found in virtual reality trials involving fear of animals, there was no significant association between sense of presence and perceived anxiety in individuals with social anxiety (Ling et al., in press).

Research has demonstrated large effect sizes for VRET for a variety of anxiety disorders (Clough & Casey, 2011; Meyerbroeker et al., 2013; Meyerbröker & Emmelkamp, 2010). Yet, most of the published trials have applied virtual reality environments that do not involve verbal interaction with virtual humans. Verbal interaction is particularly crucial with regard to the treatment of social anxiety disorder. Individuals with this disorder persistently fear embarrassment in social or performance situations, mostly involving verbal interaction with others (American Psychiatric Association, 2013). Social anxiety disorder is one of the most prevalent mental disorders, with an estimated 12-month prevalence of 12% in the United States population (Kessler et al., 2005). Several trials have assessed the efficacy of VRET for social anxiety disorder, however these have focused on fear of public speaking, which is only one of the situations that individuals with social anxiety disorder fear (Anderson et al., 2005; Anderson et al., 2013; Wallach, Safir & Bar-Zvi, 2009). In these trials the interaction between individuals and virtual humans was rather limited to a limited number of questions that the public audience would ask (such as “I don’t understand, could you explain again” Anderson et al., 2013). Powers et al. (2013) have recently published results of a study in which participants took part in both a virtual reality condition and an “*in vivo*” condition, while counterbalancing the condition order prior to participants’ assignment. The study facilitator presented a topic to be conducted three minutes later either in virtual reality and then *in vivo* or vice versa. The study facilitator then engaged participants in two 5 min conversations (i.e., one topic per condition). Participants rated their anxiety higher during virtual reality conversation than during *in vivo* conversation, whereas *in vivo* conversation was rated as more realistic than virtual reality conversation. However, the duration of social interaction (i.e., five minutes only) and the context in which the study was conducted has rather limited resemblance with the anticipated use of virtual reality conversation for therapeutic aims. The research facilitator was sitting on a couch next to the participant during the conversation, whereas virtual social conversations should rather be used for situations that resemble real world situations outside the office of the therapist.

In summary, there is lack of research examining the extent to which virtual reality conversation can be applied to treat social anxiety disorder. Against this background, we have recently developed a virtual reality exposure programme for treatment of individuals with social anxiety disorder that includes a wide variety of verbal interaction between the patient and virtual humans (Brinkman et al., 2012). The programme is based on semi-scripted dialogues related to different situations that might elicit anxiety in individuals with social anxiety disorder, such as having a job interview or buying a bra. In the current study, we aimed at assessing the extent to which such virtual social interaction can produce moderate levels of sense of presence and anxiety.

Another important aspect when considering applying VRET for mental disorders is related to its costs. The implementation of VRET is still rather a luxury of institutions with large budgets or external funding opportunities. In some cases more advanced display technology does increase the level of experienced presence, such as larger field of view (Ling et al., 2013). In other cases for example in the context of public speaking, the presence of stereoscopic viewing does not seem to be associated with sense of presence (Ling et al., 2012). However, the ability to evoke anxiety with different types of technology is less known. Accordingly, the use of low-cost, but effective virtual reality displays might prove critical in disseminating VRET for a much broader use. Consequently, an additional goal of the study was to examine whether a one-screen projection-based virtual reality display can successfully facilitate the activation of levels of sense of presence and anxiety in social virtual environments designed to trigger social anxiety. We hypothesized that exposure to virtual social interactions will elicit moderate levels of sense of presence and anxiety in both groups. Additionally, we expected that participants in the HMD condition will report higher scores of sense of presence and anxiety than participants in the one-screen projection-based display condition.

## Material and Methods

### Participants and procedure

To test our hypotheses we used a between subjects design. This design was preferred over a within subjects design to avoid carryover effects, where the first intervention might adversely influence the other. In total 43 participants took place in this experiment. However, due to missing data regarding five participants, the final sample consisted of 38 university students (32 females; mean age = 22.3, SD = 5.7) who were offered course credit for participation. Participants were randomly assigned to either a high quality HMD (Nvisor SX 60; high-resolution SXGA {1280 × 1024} stereoscopic head-mounted display; LCOS display; flock-of-bird head tracker updated at 115.200 Hz with 180° azimuth and roll, 90° elevation; 60° diagonal field of view) condition (*n* = 21 participants) or a one-screen projection-based virtual reality display condition (*n* = 22 participants) using a projector (Toshiba WX Series; Resolution: WXG {1280 × 1024}) and a projector screen (190 × 145 cm) where participants were located about two meters in front of (62° diagonal field of view). The total exposure duration was two times of up to 30 min with a break of ten minutes to prevent participants from experiencing motion sickness. Participants in both conditions were exposed to the same virtual social situations: buying clothes (for e.g., a bra) in a shop; attending a job interview; dining in a restaurant (for e.g., with a blind date or a friend); talking to strangers; or being interviewed by a journalist. These situations have recently been developed for treatment of individuals with social phobia and include semi-scripted dialogues that were controlled by research assistants (Brinkman et al., 2012). The dialogues were written with Editor3, which allowed recording of several virtual character responses as a reply on participant’s comments at each specific place in the dialogue. During the session, the research assistant controlled what the virtual human was saying by listening to participants’ responses and selecting an appropriate virtual human response or question. Both female and male virtual humans were used. The study was approved by the Institutional Review Board of the University of Amsterdam and all participants signed informed consent.

### Measures

Social anxiety symptoms were assessed with the Social Interaction Anxiety Scale (SIAS) (Mattick & Clarke, 1998). The SIAS consist of 19 items that assess the tendency to fear and avoidance of evaluation in social situations. Responses range from 0 (not at all) to 4 (extremely). The authors have reported good psychometric properties for the SIAS (Mattick & Clarke, 1998).

The social anxiety disorder module of the Structured Clinical Interview for Diagnostic and Statistical Manual of Mental Disorders (4th ed.; DSM–IV) (First et al., 1996) was applied to assess social anxiety disorder. This structured interview is considered the golden standard for assessing mental disorders formulated in the DSM.

Anxiety during exposure to virtual social worlds was assessed while asking participants to rate their anxiety level on the Subjective Unit of Discomfort (SUD) scale from zero (“no anxiety at all”) to ten (“the highest level of anxiety that I can imagine”) (Wolpe, 1990). In the current study, an average of all SUDs during the one hour exposure is reported as well as the highest SUD during this period of time.

Sense of presence was assessed following exposure with the Igroup Presence Questionnaire (IPQ) (Schubert, Friedmann & Regenbrecht, 2001). The IPQ consists of 14 items assessing sense of presence in virtual environments (e.g., “I was completely captivated by the virtual world”). Responses are provided using a 7-point Likert scale; rated from 0 (not at all) to 6 (very much). The IPQ can be used as a composite measure of sense of presence with scores ranging from 7 to 98. The IPQ has demonstrated good psychometric properties across multiple samples (Schubert, Friedmann & Regenbrecht, 2001). Finally, the authors provide online information on the mean of IPQ applied in different studies evaluation sense of presence with this instrument (http://www.igroup.org/pq/ipq/data.php). The assessed information on February 9th 2013 yielded a mean of 38.16 (SD = 17.53).

## Results

Participants in the HMD condition and the one-screen projection-based display condition were comparable in terms of age (*M* = 22.7 [SD = 3.7] and *M* = 22.0 [SD = 6.9] for the HMD condition and the one-screen projection condition, respectively; *t*(36) = 0.37, *p* = 0.71). They were further comparable regarding gender (76.5% female in the HMD condition and 81.0% female in the one-screen projection condition; Chi-square (1, 38) = 0.74, *p* = 1.0). The mean scores for the symptoms of social phobia (SIAS) were 21.7 (SD = 14.3) and 20.1 (SD = 11.2) for participants in the HMD condition and participants in the one-screen condition, respectively. A *t*-test further revealed that the two groups did not significantly differ from each other regarding the symptoms of social phobia (*t*(36) = −0.39, *p* = .70). Finally, none of the participants met criteria for social anxiety disorder according to the SCID.

The mean scores for the sense of presence (IPQ) were 55.4 (SD = 11.4) and 38.1 (SD = 14.7) for participants in the HMD condition and participants in the one-screen condition, respectively. A comparison of these data with those provided online by the authors of the IPQ indicates that both our groups reported similar levels of presence to the mean reported by Schubert and colleagues (2001) (see information on the IPQ where a mean of 38.16 [SD = 17.53] is reported). Results of a *t*-test showed that participants in the HMD condition reported significantly higher scores of sense of presence than those in the one-screen condition (*t*(41) = −4.51, *p* < .001).

In the HMD group, the mean score for the highest level of anxiety during exposure was *M* = 4.8 (SD = 2.0) and the mean score for the averaged anxiety level was 3.2 (SD = 1.8). In the one-screen condition, the mean score for the highest anxiety level was 4.8 (SD = 2.0), whereas the averaged anxiety level was 3.4 (SD = 1.8). A comparison of the conditions did not reveal any significant difference between them neither regarding the highest level of anxiety (*t*(36) = 0.04, *p* = .97) nor regarding the averaged anxiety level during the whole one hour exposure (*t*(36) = −0.35, *p* = .74).

Finally, correlation analyses were conducted to assess the association between sense of presence and self-reported anxiety during exposure to virtual worlds in both conditions. In none of the groups was sense of presence significantly related to either the highest level of anxiety (*r* = 0.29 and *r* = 0.29 for the HMD condition and one-screen condition, respectively) nor the averaged level of anxiety (*r* = 0.27 and *r* = 0.29 for the HMD condition and one-screen condition, respectively).

## Discussion

Our findings provide preliminary evidence that virtual social interactions can elicit sense of presence and anxiety in VRET. The results are in line with the findings reported by Powers et al. (2013) and expend these by examining sense of presence and perceived anxiety in virtual worlds specifically designed for psychotherapeutic use involving virtual realities with individuals with social anxiety disorder. With regard to this disorder, several trials have shown that VRET can be effectively used to treat fear of public speaking (Anderson et al., 2013; Wallach, Safir & Bar-Zvi, 2009). Our results suggest that social interaction between patients and virtual humans can be successfully implemented in VRET and consequently be used to treat all relevant aspects of social anxiety disorder that involve social interaction. Future research needs to examine the extent to which VRET involving social interaction can be as effective in treating social anxiety disorder as other efficacious interventions, such as cognitive behaviour therapy (Powers, Sigmarsson & Emmelkamp, 2008).

The current findings further suggest that one-screen projection-based displays can successfully activate anxiety in social virtual environments despite the lower sense of presence. Future research should replicate these findings with individuals with a diagnosis of social phobia or other anxiety disorders. If replicable, the findings suggest that VRET might be successfully applied via low-cost displays. This would enable the therapeutic utilization of VRET outside institutions with large budgets or external funding opportunities and thus would largely increase the number of clients benefiting from VRET. It should be noted however, that our study must be seen as a pilot examination of the extent to which one-screen projection can elevate similar levels of anxiety as compared to a head-mounted display (HMD) with motion tracker and sterescopic view condition. Future research should examine levels of anxiety among individuals with social anxiety in virtual reality one-screen projection as compared to more advanced virtual reality displays.

The results further suggest that self-reported presence is not a good indicator of the extent to which social anxiety can be activated within virtual environments. Previous research has also yielded that some level of presence is a necessary but insufficient requirement for an effective use of VRET (Krijn et al., 2004; Price & Anderson, 2007) and that the magnitude of the association between sense of presence and anxiety in VRET might depend on the disorder patients are being treated for (Ling et al., in press). With regard to social anxiety disorder, levels of subjective anxiety seem to be independent of sense of presence (Ling et al., in press). One explanation is that the used presence scale does not sufficiently capture the essential element of social presence, which might be key for virtual social interaction. Another explanation for this non-significant relationship between anxiety and sense of presence in social anxiety disorder might be explained by the presence of the therapist. Patients with social anxiety disorders might be more preoccupied with a potential negative evaluation by the therapist than patients with other anxiety disorders. Accordingly, even if patients perceive virtual worlds as little realistic and thus report a limited sense of presence, they might still feel anxious during VRET as the therapist is monitoring the session and thus might negatively evaluate the patient’s performance. Future research needs to examine the role of the therapist in sense of presence within social virtual interactions. However, it must be noted that in the current study the lack of significant correlation between sense of presence and anxiety might also be a result of the small sample sizes in each group.

The use of a non-clinical sample limits the generalization of our findings. However, the finding that individuals without social anxiety disorder reported moderate levels of anxiety in virtual social situations might indicate that these worlds are likely to invoke even higher levels of anxiety by individuals with social anxiety disorder. A further limitation of our study is the small sample sizes. Yet, these results do not offer any indication that larger sample sizes might reveal that HMD is associated with higher anxiety levels.

In summary, this study suggests that social interactions can establish sense of presence as well as elicit anxiety in VRET and that one-screen projection-based displays can successfully activate anxiety in social virtual environments.

## Supplemental Information

10.7717/peerj.337/supp-1 Supplemental Information 1 Dataset in spss format

## References

[ref-1] American Psychiatric Association (2013). Diagnostic and statistical manual of mental disorders.

[ref-2] Anderson PL, Price M, Edwards SM, Obasaju MA, Schmertz SK, Zimand E, Calamaras MR (2013). Virtual reality exposure therapy for social anxiety disorder: a randomized controlled trial. Journal of Consulting and Clinical Psychology.

[ref-3] Anderson PL, Zimand E, Hodges LF, Rothbaum BO (2005). Cognitive behavioral therapy for public-speaking anxiety using virtual reality for exposure. Depression and Anxiety.

[ref-4] Brinkman WP, Hartanto D, Kang N, de Vliegher D, Kampmann IL, Morina N, Emmelkamp PMG, Neerincx MA (2012). A virtual reality dialogue system for the treatment of social phobia. Proceedings of the 30th international conference on Human factors in computing systems (CHI’12).

[ref-5] Clough BA, Casey LM (2011). Technological adjuncts to increase adherence to therapy: a review. Clinical Psychology Review.

[ref-6] First MB, Spitzer RL, Gibbon M, Williams JBW (1996). Structured clinical interview for DSM-IV axis I disorders, clinician version (SCID-CV).

[ref-7] Kessler RC, Chiu WT, Demler O, Merikangas KR, Walters EE (2005). Prevalence, severity, and comorbidity of 12-month DSM-IV disorders in the national comorbidity survey replication (vol 62, pg 617, 2005). Archives of General Psychiatry.

[ref-8] Krijn M, Emmelkamp P, Biemond R, de Ligny C, Schuemie M, van der Mast C (2004). Treatment of acrophobia in virtual reality: the role of immersion and presence. Behaviour Research and Therapy.

[ref-9] Ling Y, Brinkman WP, Nefs HT, Qu C, Heynderickx I (2012). Effects of stereoscopic viewing on presence, anxiety, and cybersickness in a virtual reality environment for public speaking. Presence: Teleoperators and Virtual Environments.

[ref-10] Ling Y, Nefs HT, Brinkman WP, Qu C, Heynderickx I (2013). The effect of perspective on presence and space perception. PLoS ONE.

[ref-11] Ling Y, Brinkman WP, Nefs HT, Morina N, Heynderickx I A meta-analysis on the relationship between self-reported presence and anxiety in virtual reality exposure therapy for anxiety disorders. PLoS ONE.

[ref-12] Mattick R, Clarke J (1998). Development and validation of measures of social phobia scrutiny fear and social interaction anxiety. Behaviour Research and Therapy.

[ref-13] Meyerbröker K, Emmelkamp PM (2010). Virtual reality exposure therapy in anxiety disorders: a systematic review of process-and-outcome studies. Depression and Anxiety.

[ref-14] Meyerbroeker K, Morina N, Kerkhof GA, Emmelkamp PM (2013). Virtual reality exposure therapy does not provide any additional value in agoraphobic patients: a randomized controlled trial. Psychotherapy and Psychosomatics.

[ref-15] Powers MB, Briceno NF, Gresham R, Jouriles EN, Emmelkamp PMG, Smits JAJ (2013). Do conversations with virtual avatars increase feelings of social anxiety?. Journal of Anxiety Disorders.

[ref-16] Powers MB, Emmelkamp PMG (2008). Virtual reality exposure therapy for anxiety disorders: a meta-analysis. Journal of Anxiety Disorders.

[ref-17] Powers MB, Sigmarsson SR, Emmelkamp PMG (2008). A meta-analytic review of psychological treatments for social anxiety disorder. International Journal of Cognitive Therapy.

[ref-18] Price M, Anderson P (2007). The role of presence in virtual reality exposure therapy. Journal of Anxiety Disorders.

[ref-19] Schubert T, Friedmann F, Regenbrecht H (2001). The experience of presence: factor analytic insights. Presence-Teleoperators and Virtual Environments.

[ref-20] Wallach HS, Safir MP, Bar-Zvi M (2009). Virtual reality cognitive behavior therapy for public speaking anxiety A randomized clinical trial. Behavior Modification.

[ref-21] Wolpe J (1990). The practice of behavior therapy.

